# A humanized *Caenorhabditis elegans* model of hereditary spastic paraplegia-associated variants in KLC4

**DOI:** 10.1242/dmm.050076

**Published:** 2023-08-29

**Authors:** Selin Gümüşderelioğlu, Lauren Resch, Trisha Brock, Undiagnosed Diseases Network, G. W. Gant Luxton, Heidi Cope, Queenie K.-G. Tan, Christopher Hopkins, Daniel A. Starr

**Affiliations:** ^1^ Department of Molecular and Cellular Biology, University of California, Davis, Davis, CA 95616, USA; ^2^ InVivo Biosystems, Eugene, OR 97402, USA; ^3^ NIH Common Fund, Bethesda, MD 20892, USA; ^4^ Division of Medical Genetics, Department of Pediatrics, Duke University Medical Center, Durham, NC 27710, USA

**Keywords:** *Caenorhabditis elegans*, Hereditary spastic paraplegia, Kinesin light chain

## Abstract

Hereditary spastic paraplegia (HSP) is a group of degenerative neurological disorders. We identified a variant in human kinesin light chain 4 (*KLC4*) that is suspected to be associated with autosomal-dominant HSP. How this and other variants relate to pathologies is unknown. We created a humanized *Caenorhabditis elegans* model in which *klc-*2 was replaced by human *KLC4* (referred to as *hKLC4*) and assessed the extent to which *hKLC4* retained function in the worm. We observed a slight decrease in motility but no nuclear migration defects in the humanized worms, suggesting that *hKLC4* retains much of the function of *klc-2*. Five *hKLC4* variants were introduced into the humanized model. The clinical variant led to early lethality, with significant defects in nuclear migration when homozygous and a weak nuclear migration defect when heterozygous, possibly correlating with the clinical finding of late-onset HSP when the proband was heterozygous. Thus, we were able to establish humanized *C. elegans* as an animal model for HSP and to use it to test the significance of five variants of uncertain significance in the human gene *KLC4*.

## Teaser

**Summary:** We identified a variant in *KLC4* associated with hereditary spastic paraplegia. The variant had physiological relevance in a humanized *C. elegans* model in which we replaced *klc-2* with human *KLC4*.

## INTRODUCTION

Over 10,000 disorders are classified as rare diseases, each affecting fewer than 1/2000 people (Ferreira, 2019). Together, they are not rare; over 4% of the world's population is currently suffering from a rare disease (Nguengang Wakap et al., 2020). Diagnoses, let alone treatments, of rare diseases are difficult because underlying mutations are spread over 8000 genes (Ferreira, 2019). Even whole-genome sequencing leads to a definitive diagnosis only ∼25% of the time (Smedley et al., 2021). More often, a definitive diagnosis is not returned, and the clinician is left with a list of variants of uncertain significance and little idea as to which of these variants are pathogenic. Thus, one of the biggest challenges in genomic medicine is the validation of which identified variant is pathogenic. The bottleneck facing clinical geneticists is a need for functional data that can assess the pathogenicity of a variant of uncertain significance.

Hereditary spastic paraplegia (HSP) is a group of monogenetic diseases that are classified as rare diseases that present at various times throughout life. Individuals characteristically suffer from neurodegeneration in the longest motor neurons, leading to progressive spasticity and lower-limb weakness (Parodi et al., 2017; Gumeni et al., 2021; Shribman et al., 2019). Upwards of 79 genes have been linked to HSP, yet geneticists fail to obtain definitive genetic diagnoses in over half of suspected HSP individuals (Parodi et al., 2017; Gumeni et al., 2021; Shribman et al., 2019). This suggests that mutations in additional unknown genes lead to HSP. Moreover, once new candidate HSP genes are identified, we need an *in vivo* model to access the physiological significance of newly identified variants for a timely clinical diagnosis (Hopkins et al., 2022).

Functional studies *in vivo* are important for variant assessment. *Caenorhabditis elegans* is a model system that can relatively inexpensively test variants of uncertain significance at the speed needed for inclusion in a clinical report (Baldridge et al., 2021). *C. elegans* also allows examination of function in the context of a developing tissue and the use of a variety of biochemical, developmental and quantitative cellular assays needed to detect subtleties of variant biology. These advantages have led to many reports modeling human diseases in *C. elegans* (Kropp et al., 2021)*.* Thus, humanized *C. elegans* models are likely to be useful in testing the *in vivo* consequences of variants of uncertain significance identified in the clinic. Here, we report the design and use of a humanized *C. elegans* model to test the clinical significance of variants of uncertain significance in the human kinesin light chain gene identified in individuals with HSP, *KLC4*.

Molecular motor-based transport along microtubules is essential for the function and survival of eukaryotic cells (Ross et al., 2008). Microtubule motors are especially important in transporting organelles and molecules down the length of long motor neuron axons (Hirokawa et al., 2010; Perlson et al., 2010; Saxton and Hollenbeck, 2012). Disrupting motors leads to a variety of neurodegenerative diseases (Mandelkow and Mandelkow, 2002; Kurd and Saxton, 1996; Giudice et al., 2014). Kinesin-1 is the founding member of the kinesin superfamily of microtubule motors (Vale et al., 1985). Kinesin-1 consists of a tetramer of two kinesin light chains that bind to the tails of two kinesin heavy chains. The heavy chains, called KIF5B in humans, bind microtubules and provide the ATPase motor activity, whereas the light chains serve as cargo adapters (Verhey et al., 1998). In the presence of a cargo bound to the light chains, kinesin-1 is activated to move towards the plus end of microtubules. In humans, there are four different kinesin-1 light chains: KLC1, KLC2, KLC3 and KLC4. Although KLC1, KLC2 and KLC3 are relatively well studied (Rahman et al., 1998; Junco et al., 2001; Zhang et al., 2012), and their functions and how they are involved in certain diseases are known, KLC4 is understudied. Yet, mutations in *KLC4* are linked to diseases including lung cancer (Baek et al., 2018, 2020) and HSP (Bayrakli et al., 2015). The goal of this study was to further explore the link between *KLC4* and HSP by developing a humanized *C. elegans* model as a clinical avatar to test the functions of variants of uncertain significance in the gene *KLC4*.

## RESULTS

### Clinical description of an affected individual with HSP

A male individual with the clinical *KLC4* variant presented to the Undiagnosed Diseases Network (UDN) with slowly progressive myelopathy and neuropathy since ∼50[Supplementary-material sup1]years of age. Initial symptoms included numbness, followed by weakness and lower-extremity hypertonia, hyperreflexia and spasticity. The individual's symptoms worsened over the next 20[Supplementary-material sup1]years, although he maintained normal cognitive ability. Ophthalmologic evaluation showed thinning of the ganglion cell layer and papillomacular bundle, but no visual changes. Of note, he also had celiac disease, which can cause myelopathy and neuropathy, but he had been compliant with treatment with no obvious symptoms. The individual worked as an agronomist and was frequently exposed to herbicides and pesticides, potentially complicating the diagnosis.

### Selection of variants of *KLC4* to aid molecular diagnosis of individuals with HSP

A heterozygous suspected pathogenic variant was identified in the individual with HSP described above, in which a GG pair of nucleotides in the open reading frame of *KLC4* was deleted to cause a frame shift (NM_201523.2; c.1160-1161delGG; p.G369Afs*8) (Fig. S1). The predicted mutant KLC4 protein (G369fs) replaces the glycine at position 369 with an alanine, followed by eight additional novel residues and a premature stop codon that truncates more than a third of the protein. For *KLC4*, only 33% of the expected loss-of-function variants were observed (https://gnomad.broadinstitute.org/; GnomAD v2.1.1, assessed 5 June 2023), suggesting that it is under selection against loss-of-function variants (Karczewski et al., 2020). This suggests that, in a subset of genetic contexts, the gene variants can be associated with an autosomal-dominant disorder, and therefore it is considered a heterozygous variant of uncertain significance. There was no evidence of any duplication or deletion of the *KLC4* gene. The individual has an unaffected sibling who did not harbor the *KLC4* variant.

We turned to bioinformatic databases in attempts to identify four *KLC4* variants of uncertain significance, two predicted pathogenic and two predicted benign variants, as reference alleles in addition to the clinical *KLC4* G369fs variant (Fig. S1). Two missense *KLC4* mutations, R72H and R358H, were chosen as predicted benign controls. R72H was observed at a very-high frequency (6803×) in unaffected populations using GnomAD (Karczewski et al., 2020). R358H was seen at 9× in GnomAD and was scored as possibly damaging in PolyPhen-2 (Adzhubei et al., 2010), tolerated in Sorting Intolerant From Tolerant (SIFT; Sim et al., 2012), and neutral in Combined Annotation-Dependent Depletion (CADD; Kircher et al., 2014) and Rare Exome Variant Ensemble Learner (REVEL; Ioannidis et al., 2016). Two other missense mutations were chosen because they were predicted to be pathogenic variants. Both T381I and A295P were identified as damaging by PolyPhen-2 (Adzhubei et al., 2010) and possibly damaging by SIFT (Sim et al., 2012). Thus, we obtained a collection of five *KLC4* mutations for testing in an *in vivo* model.

### A *C. elegans* model in which human *KLC4* rescues the lethality of a *klc-2* null allele

**Fig. 1.**
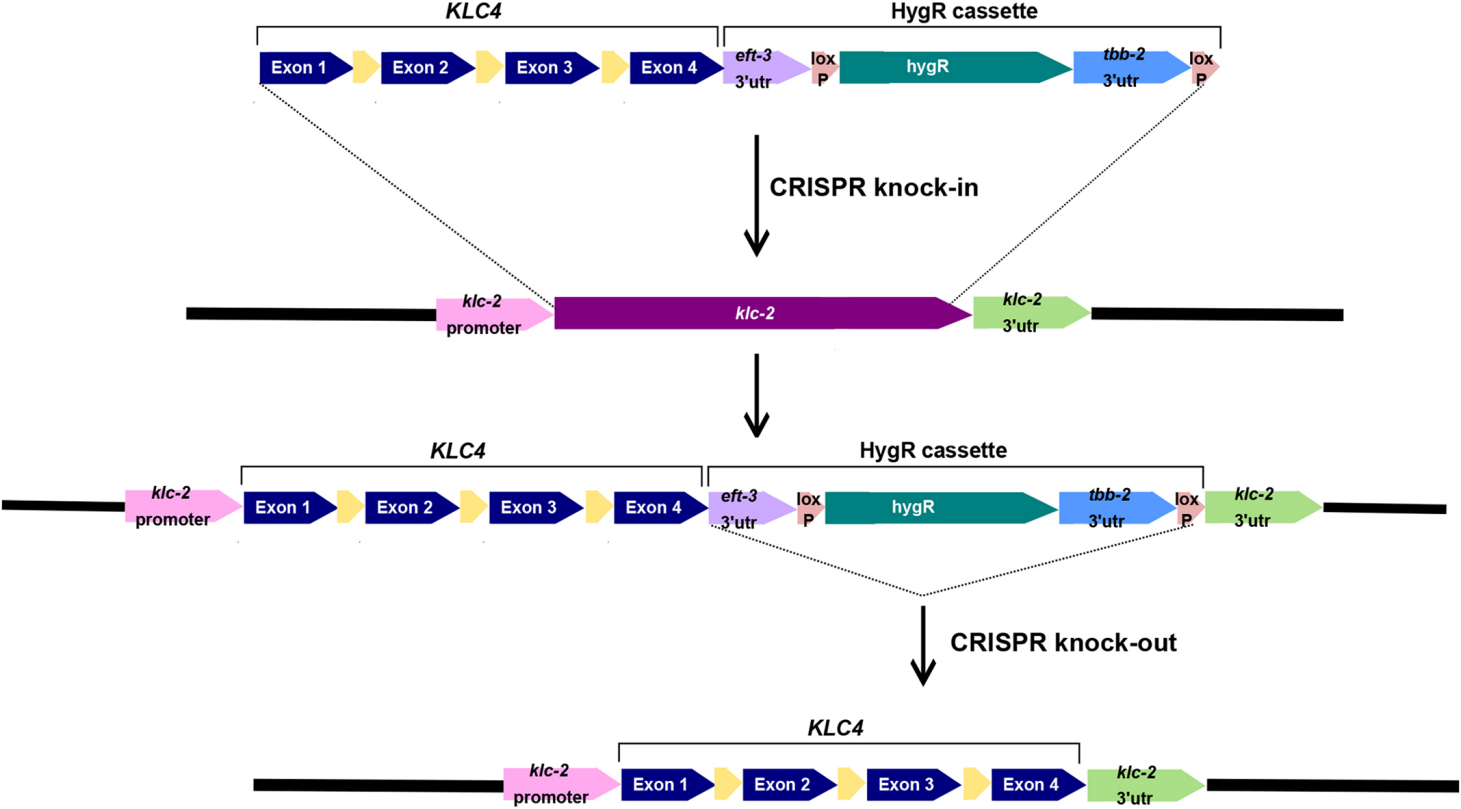
**The CRISPR/Cas9-mediated genome-editing workflow used to generate *hKLC4* worms.** The inserted sequence contains human gene *KLC4* (exons shown in blue; synthetic introns shown in yellow) and hygromycin-resistant gene selection cassette (HygR). See text for details. utr, untranslated region.

**Fig. 2.**
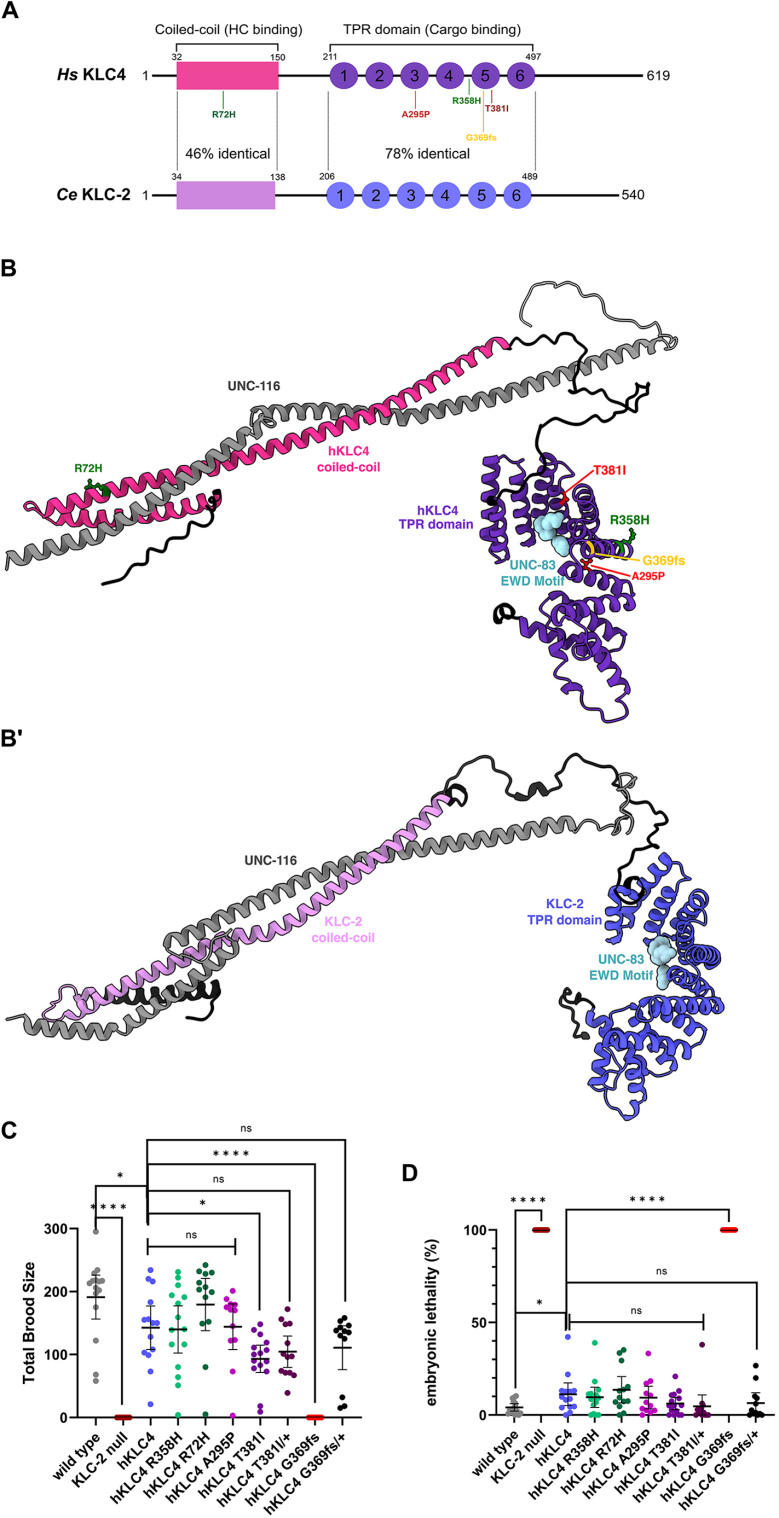
**A *C. elegans* model in which human *KLC4* replaces *klc-2*.** (A) Illustration of the human KLC4 and *C. elegans* KLC-2 proteins. The predicted coiled-coil domain (pink) that binds to the kinesin heavy chain and six tetratricopeptide (TPR) repeats (purple) that function together to bind cargo are shown for both proteins. The coiled-coil regions of KLC4 and KLC-2 are 46% identical and the TPR domains are 78% identical. The missense mutations used in this study are shown. Green mutants are predicted benign, red mutants are predicted pathogenic, and the yellow mutant is the clinical variant of uncertain significance. *Hs*, *Homo sapiens*; *Ce*, *Caenorhabditis elegans*. (B) AlphaFold prediction of the wild-type structures and interaction between hKLC4 (coiled-coil domain in pink and TPR domain in purple), the kinesin heavy chain UNC-116 (gray) and UNC-83 (light blue). Where the missense mutations used in this study would lie in the wild-type structures is shown. Green mutants are predicted benign, red mutants are predicted pathogenic, and the yellow mutant is the clinical variant of uncertain significance. (B′) AlphaFold prediction of the structures and interaction between wild-type KLC-2 (coiled-coil domain in lavender and TPR domain in dark blue), the kinesin heavy chain UNC-116 (gray) and UNC-83 (light blue). (C) Quantification of the total brood size of *C. elegans* strains. (D) Quantification of the embryonic lethality (%) of *C. elegans* strains. For C and D, each data point represents one animal. *n*=10-15 for each strain. Means with 95% confidence intervals are shown in error bars. Unpaired two-tailed Student’s *t*-tests were performed on the indicated comparisons. ns, not significant (*P*>0.05); *P<0.05; ****P<0.0001.

We aimed to make a humanized *C. elegans* model to test the physiological significance of *KLC4* mutations (Fig. 1). Kinesin-1 plays similar important roles in *C. elegans* as it does in humans, including moving synaptic vesicles in motor neurons and nuclei in hypodermal precursors (Meyerzon et al., 2009; Sakamoto et al., 2005). The *C. elegans* genome encodes two kinesin light chains, KLC-1 and KLC-2 (Koushika and Nonet, 2000). Although they act redundantly in the *C. elegans* embryo during the pre-anaphase translocation of the meiotic spindle (Yang et al., 2005), KLC-2 is likely to be the primary light chain for kinesin-1 in most of development. Compared with KLC-1, KLC-2 has a higher level of sequence homology to other metazoan kinesin light chains. KLC-2 also directly interacts with the kinesin heavy chain (UNC-116) to form a kinesin-1 complex, and null alleles of *klc-2* cause severe larval lethality (Sakamoto et al., 2005). Human KLC4 and *C. elegans* KLC-2 proteins both have a predicted coiled-coil domain that binds to the kinesin heavy chain and six tetratricopeptide (TPR) repeats that function together to bind cargo (Fig. 2A-B′). The coiled-coil regions of KLC4 and KLC-2 are 46% identical and the TPR domains are 78% identical (Fig. 2A; Fig. S1). Using AlphaFold (Jumper et al., 2021), we were able to model the predicted structures of KLC-2 and KLC4 as well as their interactions with UNC-116 and the *C. elegans* protein UNC-83 that acts as a binding adaptor for kinesin-1 (Meyerzon et al., 2009; Taiber et al., 2022). In addition to the evolutionarily conserved sequences the two proteins share, we saw that KLC-2 and KLC4 share a high level of similarity in their structural features and how they interact with the *C. elegans* proteins UNC-116 and UNC-83. Thus, we chose the *klc-2* locus to engineer in human *KLC4* to make a humanized model. Human KLC4 has a major isoform encoding a 619 residue protein that is expressed more broadly and at higher levels than the 637 residue isoform, according to the Transcript Support Level (TSL) method (Yates et al., 2016). Therefore, we used the shorter 619 residue protein isoform to make a humanized *C. elegans* line. A humanized *C. elegans* model was generated in which the endogenous *klc-2* gene was replaced by human *KLC4* using CRISPR/Cas9 genome editing (Fig. 1). The coding region for human *KLC4* was codon optimized, and placed under control of the endogenous *klc-2* promoter and the 5′ and 3′ untranslated regions (UTRs) of the *klc-2* gene. In addition, three synthetic *C. elegans* introns were inserted into *KLC4* to maximize its expression in *C. elegans* (Fig. 1).

After generating the humanized *C. elegans* line in which human *KLC4* replaced *klc-*2 (hereafter referred to as the *hKLC4* line), we compared the fitness of the new model to wild type. We quantified the viability of the *hKLC4* line by measuring its brood size and embryonic lethality in comparison to wild type (Fig. 2C,D). The *hKLC4* strain was viable as a homozygous strain, while *klc-2* null animals are 100% embryonic or L1 larval lethal, suggesting that *hKLC4* rescued many of the essential *klc-2* functions. However, the *hKLC4* line had significant levels of embryonic lethality (11.2±5.4% compared to 4.1±1.8% in wild type; mean±95% confidence intervals) and a slightly lower brood size (142.6±30.2 compared to 191.1±31.9 in wild type) (Fig. 2C,D), suggesting that *hKLC4* animals were not as fit as wild type, perhaps because *C. elegans* were maintained at ∼22°C instead of the 37°C that the human protein has evolved to have optimal activity at. Nonetheless, most animals with only human KLC4 in place of endogenous KLC-2 were quite viable, fertile and appeared relatively normal, suggesting that the *hKLC4* model would be of use as a clinical avatar.

**Fig. 3.**
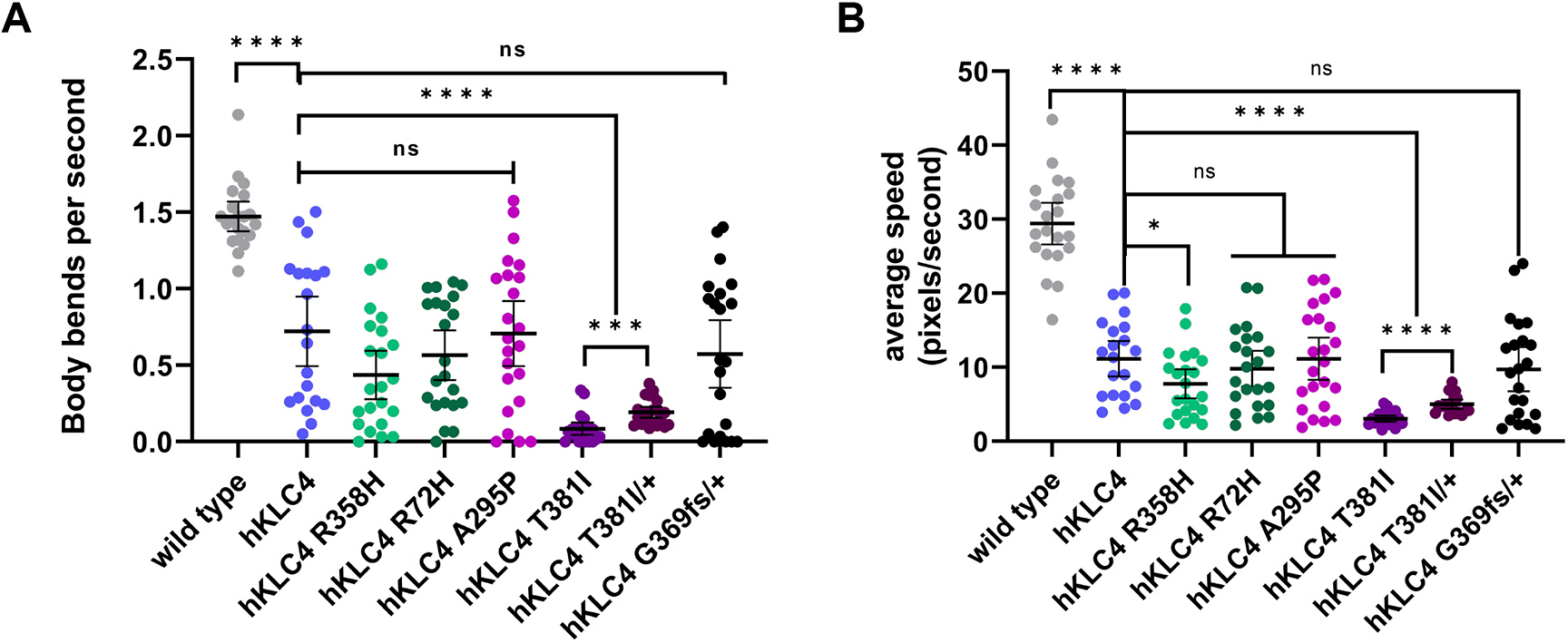
***hKLC4* worms have a motility defect that is enhanced by the predicted pathogenic mutation T381I.** (A) Quantification of *C. elegans* swimming by counting the number of body bends per second. (B) Quantification of *C. elegans* swimming by average speed in pixels/s. For A and B, each point represents one L4-stage animal. *n*=20 for each strain. Means with 95% confidence intervals are shown in error bars. Unpaired two-tailed Student’s *t*-tests were performed on the indicated comparisons. ns, not significant (*P*>0.05); *P<0.05; ****P<0.0001.

The *hKLC4* animals had no obvious phenotypes affecting their ability to crawl on the surface of an agar plate. However, swimming and crawling are two different forms of movement in terms of their kinematics and muscle activity (Pierce-Shimomura et al., 2008). Therefore, to conduct a more comprehensive movement analysis of the humanized animals, we used a swimming assay in which we observed head thrashing in liquid. We scored swimming by measuring the number of body bends per second (BBPS) and average speed per animal to assess the motility of *hKLC4* animals (Pierce-Shimomura et al., 2008; Mattout et al., 2011). We observed that *hKLC4* animals had a significantly lower number of BBPS and average speed than wild-type worms (0.72±0.21 BBPS compared to 1.48±0.09 BBPS in wild type, and 11.16±2.24 pixels/s compared to 29.43±2.64 pixels/s in wild type; mean±95% confidence intervals) (Fig. 3A,B; Movies 1 and 2). The twofold effect in BBPS and almost threefold effect in the average speed suggest that the humanized line has a significant motility defect.

**Fig. 4.**
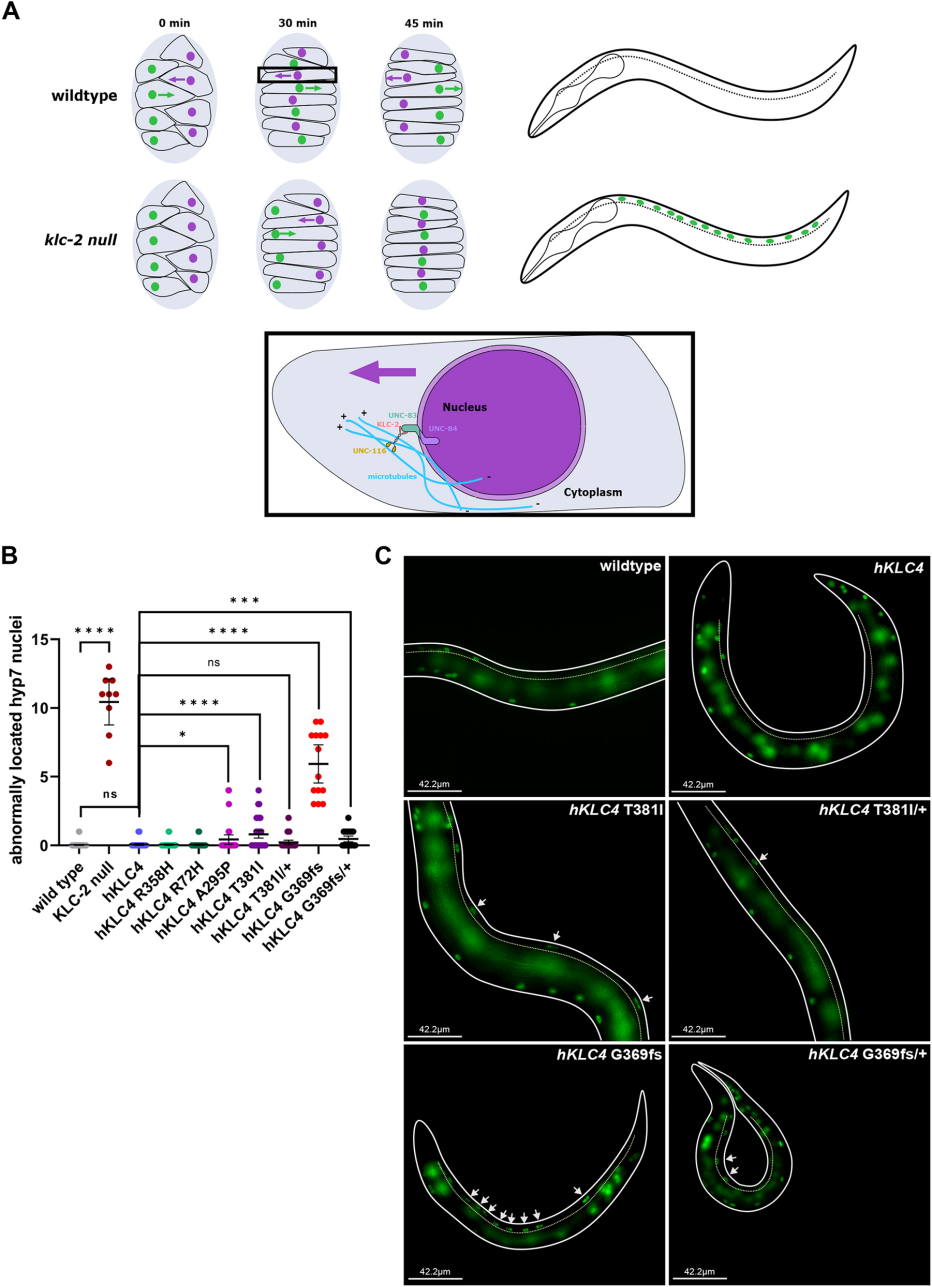
**The clinical variant of uncertain significance *hKLC4* G369fs causes a severe hyp7 nuclear migration defect.** (A) Illustration of the dorsal view of hyp7 nuclear migration during mid-embryogenesis in wild-type and *klc-2* null embryos (top left) and illustration of the lateral view of *C. elegans* larvae (dotted lines mark the dorsal side of the animals) (top right). At *t*=0 min, nuclei of hyp7 precursors (green and purple) are found on the right and left sides of the dorsal surface of both embryos. At *t*=30 min, in wild-type embryos, the nuclei, mediated by the LINC complex (UNC-83 and UNC-84), intercalate to form a single row spanning the dorsal midline. In *klc-2* null embryos, nuclear migration is delayed and hyp7 nuclei are still on the right and left sides of the dorsal surface, and the precursor cells have already started to elongate as they do in wild-type embryos. At *t*=45 min, wild-type nuclei migrate contralaterally toward the plus ends of microtubules (blue; bottom) across the dorsal midline to the opposite side of the embryo, and the *klc-2* null nuclei reach the dorsal midline and stop moving after that. In the larvae, no hyp7 nuclei are found at the dorsal cord of the wild-type animals and up to 16 hyp7 nuclei can be found at the dorsal cord of *klc-2* null animals. The LINC complex, consisting of the KASH protein UNC-83 at the outer nuclear membrane and the SUN protein UNC-84 at the inner nuclear membrane, recruits kinesin-1 to the surface of nuclei through binding to KLC-2 and transmits the forces to inside the nucleus. (B) Quantification of failed hyp7 nuclear migration via counting the number of abnormally located (at the dorsal cord) nuclei. Each point represents the total number of abnormally located hyp7 nuclei per animal. *n*=9 for KLC-2 null; *n*=14 for hKLC4 G369fs; *n*=20 for all the other strains. Means with 95% confidence intervals are shown in error bars. Unpaired two-tailed Student’s *t*-tests were performed on the indicated comparisons. ns, not significant (*P*>0.05); *P<0.05; ***P<0.001; ****P<0.0001. (C) Lateral view of L4 wild type, *hKLC4*, *hKLC4* T381I and *hKLC4* T381I/+, and L1-early L2 *hKLC4* G369fs and *hKLC4* G369fs/+ animals expressing hypodermal nuclear GFP. Dotted lines mark the dorsal side of the animal. Arrows show abnormally located (in the dorsal cord) hyp7 nuclei. Scale bars: 42.2 µm.

A second *klc-2*-dependent assay examined nuclear migration (Fridolfsson et al., 2018). There is no known link between nuclear migration and HSP, but because interkinetic nuclear migration is important for neurodevelopment and defects lead to disease (Bone and Starr, 2016), and we had an assay that is dependent on *klc-2* (Meyerzon et al., 2009), we reasoned that this assay could be used to study the function of the *hKLC4* variants. In mid-embryogenesis, two rows of hyp7 precursors on the dorsal surface of embryos intercalate to form a single row spanning the dorsal midline. Next, nuclei migrate contralaterally toward the plus ends of microtubules across the dorsal midline to the opposite side of the embryo (Sulston et al., 1983) (Fig. 4A). Successful completion of nuclear migration in embryonic hyp7 hypodermal cells requires kinesin-1 heavy chain and KLC-2 (Meyerzon et al., 2009). The linker of nucleoskeleton and cytoskeleton (LINC) complex, consisting of the Klarsicht/ANC-1/SYNE homology (KASH) protein UNC-83 at the outer nuclear membrane and the Sad1/UNC-84 (SUN) protein UNC-84 at the inner nuclear membrane, recruits kinesin-1 to the surface of nuclei and transmits the forces to inside the nucleus (Starr, 2019) (Fig. 4A). Null mutant *klc-2(km28)* larvae that barely escape embryonic lethality had an average of 10.4±1.4 hyp7 nuclei abnormally located in the dorsal cord, compared to 0.07±0.08 in wild type (Fig. 4B). We have previously shown that this represents a nearly penetrant nuclear migration defect (Fridolfsson and Starr, 2010) and that *klc-2(km28)* is a likely null allele resulting from a 0.6 kb deletion of parts of exons 2-4 (Sakamoto et al., 2005). To analyze whether KLC4 was able to retain KLC-2 function in the humanized worms, we counted the number of hyp7 nuclei abnormally present in the dorsal cord. The *hKLC4* animals had no significant nuclear migration defects compared to wild type (Fig. 4B,C). Together, these data suggest that *hKLC4* can substitute for most of the function of *klc-2* in *C. elegans*.

### Functional analysis of *hKLC4* variants of uncertain significance

After showing that the *hKLC4* animals were healthy, our goal was to introduce missense variants into the *hKLC4* avatar to test their possible effects on *C. elegans* development as an indicator of clinical interest. Four *hKLC4* variants were introduced into our *hKLC4* worm line using CRISPR/Cas9 gene editing, chosen as discussed above. Variants *hKLC4* R72H and *hKLC4* R358H were predicted to be benign, and *hKLC4* A295P and *hKLC4* T381I were predicted to be pathogenic (Fig. S1). We quantified the brood size and embryonic lethality of the missense variants and found that neither the predicted benign nor the predicted pathogenic *hKLC4* mutations had a significantly deleterious effect on the percentage embryonic lethality observed in the parental *hKLC4* line (Fig. 2D). However, one of the predicted pathogenic mutations, *hKLC4* T381I, led to a significant decrease in the brood size (Fig. 2C).

We had similar results when we observed the swimming behavior of the missense mutants. The two predicted benign variants and the A295P variant had no effect on the swimming rate of the *hKLC4* parental line. However, the predicted disease allele *hKLC4* T381I caused a severe motility defect, with only 0.085±0.038 BBPS and 3.07±0.38 pixels/s average speed (Movie 3), compared to the parental *hKLC4* worms that had 0.72±0.21 BBPS and 11.16±2.24 pixels/s average speed (Fig. 3). After seeing such severe motility defects in the *hKLC4* T381I homozygous animals, to further analyze the characteristics of this particular point mutation, we generated *hKLC4* T381I heterozygous (*hKLC4* T381I/+) animals by mating *hKLC4* T381I homozygous hermaphrodites with *hKLC4* homozygous males. Although there was a statistically significant improvement in terms of motility compared to the *hKLC4* T381I homozygous animals, *hKLC4* T381I/+ animals still had a severe motility defect (Movie 4), with 0.19±0.038 BBPS and 5.04±0.59 pixels/s average speed (Fig. 3). We also observed no statistically significant change in the total brood size and embryonic lethality of the heterozygous animals compared to those of the homozygous animals or the *hKLC4* starting strain (Fig. 2C,D).

In our nuclear migration assay, the benign variants *hKLC4* R72H and R358H did not cause any hyp7 nuclear migration defects (Fig. 4). In contrast, both predicted pathogenic variants, *hKLC4* A295P and T381I caused mild, but significant, nuclear migration defects, with 0.43±0.34 and 0.62±0.24 hyp7 nuclei observed in the dorsal cord of an average worm (Fig. 4B,C). The *hKLC4* T381I/+ animals did not have any hyp7 nuclear migration defects (0.22±0.16 hyp7 nuclei located abnormally in the dorsal cord).

### Clinical variant *hKLC4* G369fs animals have severe defects

Finally, we introduced the clinical variant of uncertain significance *hKLC4* G369fs using CRISPR/Cas9 genome editing. Attempts to generate a homozygote line failed so we suspected this variant to be lethal. We introduced G369fs into a strain humanized for *hKLC4* that also contained a translational fusion *klc-2::gfp* rescue array. The rescuing array is expressed from an extrachromosomal array that in *C. elegans* is lost in a high percentage of animals during early embryonic cell divisions (Sakamoto et al., 2005). Thus, this strain produces *hKLC4* G369fs animals both with and without the rescuing array. All *hKLC4* G369fs animals that survived to adulthood maintained the *klc-2::gfp* rescuing array, suggesting that all the animals that lost the rescuing array died as embryos or early larvae. Therefore, animals homozygous for *hKLC4* G369fs have a total brood size of zero and 100% embryonic lethality (Fig. 2C,D). We were therefore unable to measure the swimming ability of the *hKLC4* G369fs animals. However, we were able to observe nuclear migration defects in the rare *hKLC4* G369fs animals that escaped embryonic lethality and could be scored as young larvae before dying. The *hKLC4* G369fs animals had severe nuclear migration defects with a mean of 5.9±1.3 hyp7 nuclei in the dorsal cord of an animal, compared to nearly zero nuclei in the dorsal cord of the *hKLC4* animals (Fig. 4B). Taken together, these data show that the G369fs mutation is very severe, making the hKLC4 animals very sick.

To test the extent to which the *KLC4* frameshift variant at residue 369 acts in a haplo-insufficient manner or whether it is the sole contributor to the clinical features, we crossed the homozygous truncation mutant to the *hKLC4* strain to assay heterozygotes. Heterozygous *hKLC4* G369fs/+ animals were healthy and viable, with no significantly deleterious effect on the total brood size and embryonic lethality observed in the parental *hKLC4* line (Fig. 2C,D). The heterozygous *hKLC4* G369fs/+ animals also did not have a significant swimming defect (Movie 5) compared to *hKLC4* animals (Fig. 3A). However, the *hKLC4* G369fs/+ heterozygotes did have a weak, but significant, nuclear migration defect in hyp7 precursors (Fig. 4B,C). This phenotype was similar to the one observed in predicted pathogenic variants *hKLC4* A295P and *hKLC4* T381I. Thus, although heterozygous *hKLC4* G369fs/+ animals are healthier than the homozygous truncation animals, they still have a significant hyp7 nuclear migration defect.

## DISCUSSION

In this study, we characterized a heterozygous *KLC4* variant of uncertain significance detected in an individual with HSP. We engineered and used a humanized *C. elegans* model to test the physiological relevance of the variant in a heterologous *in vivo* system. We demonstrated that *C. elegans* can be used to model disease-associated variants of human *KLC4*, that we can use the *hKLC4 C. elegans* strain generated here to test the physiological impact of other *KLC4* variants, and that this strategy could be used to model neuromuscular diseases in other genes with clear orthologs in *C. elegans*, including LINC complexes that target kinesin light chains to the nucleus.

There are thousands of rare diseases, each of which affect fewer than 1/2000 people. However, combined together, ∼300-400 million people suffer from rare diseases worldwide (Nguengang Wakap et al., 2020). Whole-genome sequencing has aided in the diagnosis of rare diseases, but the identification of a genetic underpinning of a disease still fails up to 75% of the time (Smedley et al., 2021). We therefore need to develop animal models to test variants of uncertain significance to make more efficient and better clinical diagnoses. One goal of the UDN is to bring together clinicians and basic scientists to test variants of uncertain significance in animal models (Baldridge et al., 2021). Here, we report clinical findings implicating a novel variant in the kinesin light chain gene *KLC4* from a proband with HSP and the subsequent generation of a humanized *C. elegans* model to test the significance of the variant.

We identified an individual with late-onset HSP with a heterozygous variant in *KLC4* predicted to cause a frame shift at residue 369, closely followed by a premature stop codon. An additional family was previously reported where a premature stop codon after residue 277 of *KLC4* caused HSP in an autosomal-recessive manner; heterozygous family members did not have any symptoms (Bayrakli et al., 2015). Truncations in *KLC4* after either 277 or 369 residues are predicted to disrupt the TPR domain, which mediates the interaction between kinesin and the cargo adaptor (Pernigo et al., 2013; Zhu et al., 2012), suggesting that both *KLC4* variants should produce similar pathologies. The truncated KLC4 protein variant could conceivably be acting in a dominant-negative manner in the proband, which would not be recapitulated in *C. elegans* owing to likely nonsense-mediated decay of the transcript. Alternatively, there could be other protein variants in the proband acting synergistically with the truncated KLC4 protein to produce a phenotype. Variants in other genes were identified by whole-genome sequencing of the individual, and studies are underway to determine their contributions to pathogenicity.

We aimed to make a humanized *C. elegans* model to test clinical *KLC4* variants. The open reading frame of the *C. elegans* ortholog *klc-2* was successfully replaced by the human *KLC4* coding sequence. Although the *klc-2* null alleles are not viable (Sakamoto et al., 2005), the *hKLC4* model had only low levels of embryonic lethality and a slightly reduced brood size. However, the *hKLC4* animals had significant swimming defects. These could be due to defects in vesicle transport in motor neuron axons, which is a process that *klc-2* is known to be required for (Sakamoto et al., 2005). Thus, the humanized model expressing *KLC4* under control of the endogenous *klc-2* locus retained much, but not all, of the function of *klc-2*. We used these phenotypes as a baseline to compare with the effects caused by the introduction of variants of uncertain significance into the *hKLC4* line, including the clinical truncation variant. One of the four variants of uncertain significance with single amino acid changes, *hKLC4* T381I, which was predicted to be possibly pathogenic, disrupted the function of *hKLC4* in *C. elegans*. The T381I variant had phenotypes including a reduced brood size, slower thrashing in our swimming assay and an increased number of hyp7 precursor nuclei that failed to migrate. The heterozygous *hKLC4* T381I/+ animals still had a severe motility defect, but no nuclear migration defects and no statistically significant change in their brood size or embryonic lethality compared to the homozygous T381I animals. These results indicate that while even a single copy of *hKLC4* T381I is sufficient to cause a severe motility defect, a single copy of wild-type *hKLC4* is sufficient to rescue the mild hyp7 nuclear migration defect. These data suggest that the T381I variant is also likely to disrupt KLC4 function in human cells and is, therefore, likely to be a pathogenic variant as predicted by PolyPhen-2 (AdzhubeIvan et al., 2010) and SIFT (Sim et al., 2012). However, the other three tested variants are unlikely to be pathogenic. Thus, these results support the bioinformatic predictions of significance made for the *KLC4* mutations, R72H and R358H, which were predicted benign by SIFT (Sim et al., 2012), CADD (Kircher et al., 2014) and REVEL (Ioannidis et al., 2016), while not for the mutation A295P, which was identified as damaging by PolyPhen-2 (AdzhubeIvan et al., 2010) and possibly damaging by SIFT (Sim et al., 2012).

The clinical variant was homozygous lethal, and escaper larvae had severe nuclear migration defects like those with *klc-2* null alleles (Fridolfsson et al., 2010), suggesting that the clinical variant would lead to severe pathologies when homozygous. We next examined whether the *KLC4* frameshift variant at residue 369 acts in a haplo-insufficient manner. We observed a mild nuclear migration defect in heterozygous animals, consistent with other disease-associated variants. This mild phenotype in *C. elegans* could therefore be useful in predicting whether a variant of uncertain significance might cause clinical symptoms of HSP. Further work is needed to determine whether a heterozygous loss-of-function *KLC4* variant can cause HSP; this includes finding more affected individuals who harbor disease-causing *KLC4* variants.

The kinesin light chain is part of a network of proteins conserved from *C. elegans* to humans including the LINC complex-forming KASH and SUN proteins (Starr, 2019; Fridolfsson and Starr, 2010; Roux et al., 2009). Variants in the genes encoding components of human LINC complexes, including Nesprins, have been implicated in a wide variety of diseases, including neurological disorders, muscular dystrophies and various cancers (Janin et al., 2017). Humanized *C. elegans* strains for LINC complex components would provide additional reagents to test clinical variants in this important complex. The success of the humanized *KLC4 C. elegans* line described here suggests that this approach could be feasible for modeling other neuromuscular diseases associated with LINC complex dysfunction.

## MATERIALS AND METHODS

### *C. elegans* genetics and humanized strain generation

*C. elegans* strains were maintained on nematode growth medium plates seeded with OP50 *Escherichia coli* at room temperature; the N2 strain was used as wild type (Brenner, 1974). Some strains were obtained from the Caenorhabditis Genetics Center, funded by the National Institutes of Health Office of Research Infrastructure Programs (P40 OD010440). All strains were maintained, and phenotypic assays were performed at room temperature (∼22°C). The *ycIs9 I* strain, which was used to mark hypodermal nuclei with GFP, was generated along with *ycIs10 V* as previously described (Bone et al., 2014). The strains used in this study are listed in Table S1.

The *hKLC4* strain was made utilizing the transgenesis services of InVivo Biosystems. To generate the *hKLC4* strain, the coding region of the human *KLC4* open reading frame was synthesized from the ATG to the stop codon of the most supported (Transcription Support Level 1) KLC4 isoform in Ensembl (ENST00000347162.10) and placed into a plasmid, pUC57 (Guo et al., 2014). The resulting gene coding for 619 amino acids was codon optimized for *C. elegans* (Mitreva et al., 2006), and synthetic introns, which have been shown to be essential for normal expression in *C. elegans* (Blumenthal, 2012), were inserted. The sequence of this gene block is shown in Fig. S2. The *hKLC4* coding sequence was then flanked by 500 bp endogenous *C. elegans klc-2* 5′ and 3′ sequences from genomic DNA by PCR and Gibson assembly to create pNU2756. A hygromycin-resistant gene with a *tbb-2* 3′ UTR selection cassette flanked by *loxP* sites was included in pNU2756 to aid in identifying transgenic animals. We also included the 3′ UTR sequence of *eft-3* (also known as *eef-1A.1*) after the *hKLC4* stop codon because sometimes a strong 3′ UTR is needed for optimal expression (Chen et al., 2013). pNU2756 was used as the repair template for CRISPR/Cas9 genome editing (Fig. 1). Two sgRNAs targeting each end of the *klc-2* open reading frame were used to guide CRISPR/Cas9 to cut out the coding region of *klc-2* (Table S2). The sgRNAs that were preassembled with crRNA, Cas9 protein and the assembled ribonucleoprotein complex, and the repair template were then injected into the gonads of *C. elegans* young adults (Paix et al., 2015; Farboud et al., 2019). Progeny were screened for incorporation of *hKLC4* into the worm genome by selecting for the animals that could survive upon hygromycin treatment. Two strains with *hKLC4::eft-3* 3′ UTR and the hygromycin selection cassette were obtained and backcrossed to N2 wild type to minimize off-target effects. Expression of *hKLC4* was confirmed by reverse transcription quantitative PCR. Finally, using new sgRNAs (Table S2), the *hygromycin::tbb-2* 3′ UTR cassette and the *eft-3* 3′ UTR were removed, restoring the native *klc-2* 3′ UTR and generating the humanized *klc-2(knu1031[hkLC4])* strain, referred to as *hKLC4* (Fig. 1).

Point mutations were introduced into the *hKLC4* line using CRISPR/Cas9 gene editing (Farboud et al., 2019). The sgRNA and ssDNA repair template sequences used to introduce missense mutations studied in this work are listed in Table S2. To identify successfully injected animals, co-CRISPR with templates to create *dpy-10(gof)* alleles was performed (Arribere et al., 2014). Point mutants were then screened for with genomic PCR followed by restriction digest analysis. Newly generated alleles were backcrossed to N2.

To generate the humanized allele with the clinical variant of uncertain significance, *klc-2(knu1102[hKLC4(G369fs)])*, a wild-type *klc-2::gfp* translational fusion expressed from an extrachromosomal array (Sakamoto et al., 2005) was crossed into the *hKLC4* strain. Then, CRISPR/Cas9 gene editing was used as described above to introduce the mutation into *hKLC4*. By having a translational *klc-2::gfp* rescuing array, the worms were able to carry homozygous variants in *hKLC4*, even if they were not functional. To further analyze the clinical variant for haploinsufficiency, heterozygous *hKLC4* G369fs/+ animals were generated by mating *hKLC4* males with *hKLC4* G369fs*, klc-2::gfp* hermaphrodites and selecting for animals without the *klc-2::gfp* extrachromosomal array.

### AlphaFold modeling

The atomic models for the protein complex were generated using ′AlphaFold2-multimer-v2′ via ColabFold v1.3.0 (Mirdita et al., 2022) with default settings without using structure templates. Five models were generated and subsequently relaxed using amber force fields. The top-ranking model was used for Fig. 2B,B′. For visualization, some residues within the low-confidence linker region in KLC4 and KLC-2 were straightened by altering the torsion angles (phi and psi) to approximately −135° and 135°, respectively, using the ‘torsion’ command in ChimeraX (Pettersen et al., 2021).

### Clinical methods

Informed consent was obtained from the participant to participate in the National Institutes of Health (NIH)-Undiagnosed Diseases Network (UDN) protocol (15-HG-0130). Proband-only genome sequencing was performed through the UDN. Clinical investigation was conducted according to the principles expressed in the Declaration of Helsinki.

### Phenotypic assays and statistical evaluations

Brood size assays were conducted to quantify the brood size and embryonic lethality of each viable homozygous strain used in this study. For brood size assays, starting at the L4 stage, 10-15 single animals of each genotype were transferred onto fresh OP50 *E. coli* plates, labeled as Day 1, and kept at room temperature (∼22°C) for 42 h so that they became adults and had ∼24 h to lay eggs. At 42 h, adult worms were moved to new plates, labeled as Day 2, and kept at room temperature for 24 h. At the 24-h mark, adult worms were moved to new plates, labeled as Day 3, and dead eggs and young worms from the Day 1 plates were counted. At the next 24-h mark, the adult worms on the Day 3 plates were killed, and dead eggs and young worms from Day 2 plates were counted. At the next and final 24-h mark, dead eggs and young worms from Day 3 plates were counted. The total number of viable worms and dead eggs counted over the 3-day time course were plotted as the total brood size for each worm (Fig. 2C). Simultaneously, the total number of dead eggs counted over the 3-day time course was divided by the total brood size to calculate the embryonic lethality percentage for each worm and plotted (Fig. 2D).

Motilities of the strains used in this study were quantified by performing swimming assays. For the swimming assays, eight to ten L4 stage animals were put into a plate and flooded with M9 buffer. We observed and filmed worms swimming in buffer for 30 s. Using the Fiji wrMTrck plugin (Nussbaum-Krammer et al., 2015), we measured the average speed and BBPS to quantify the overall motility of the animals. Only worms that were consecutively tracked for 30 s were included in the analysis.

To quantify nuclear migration, humanized *hKLC4* strains were crossed into a GFP nuclear marker expressed in larval hypodermal nuclei (*ycIs9 I*; Table S1). Nuclear migration assays were performed as described (Fridolfsson et al., 2018). Briefly, larval worms with GFP-marked hypodermal nuclei were picked and mounted on 2% agarose pads in ∼5 µl of 1 mM tetramisole in M9 buffer. Syncytial hyp7 nuclei were scored as abnormally located if they were in the dorsal cord.

GraphPad Prism version 9.0 software was used for the statistical analyses. All the data from nuclear migration, brood size and embryonic lethality, and swimming assays were displayed as scatter plots with means and 95% confidence intervals as error bars. Sample sizes and the statistical tests are indicated in the figure legends. Unpaired two-tailed Student’s *t*-tests were performed on the indicated comparisons.

## Supplementary Material

10.1242/dmm.050076_sup1 Supplementary information
