# Supplementary material for: A humanized Caenorhabditis elegans model of hereditary spastic paraplegia-associated variants in KLC4
Source: Dis Model Mech. 2023 Aug 29;16(8):dmm050076. doi: 10.1242/dmm.050076 (PMC10481945; doi:10.1242/dmm.050076)
Supplement: Supplementary information [file dmm-16-050076-s1.pdf]

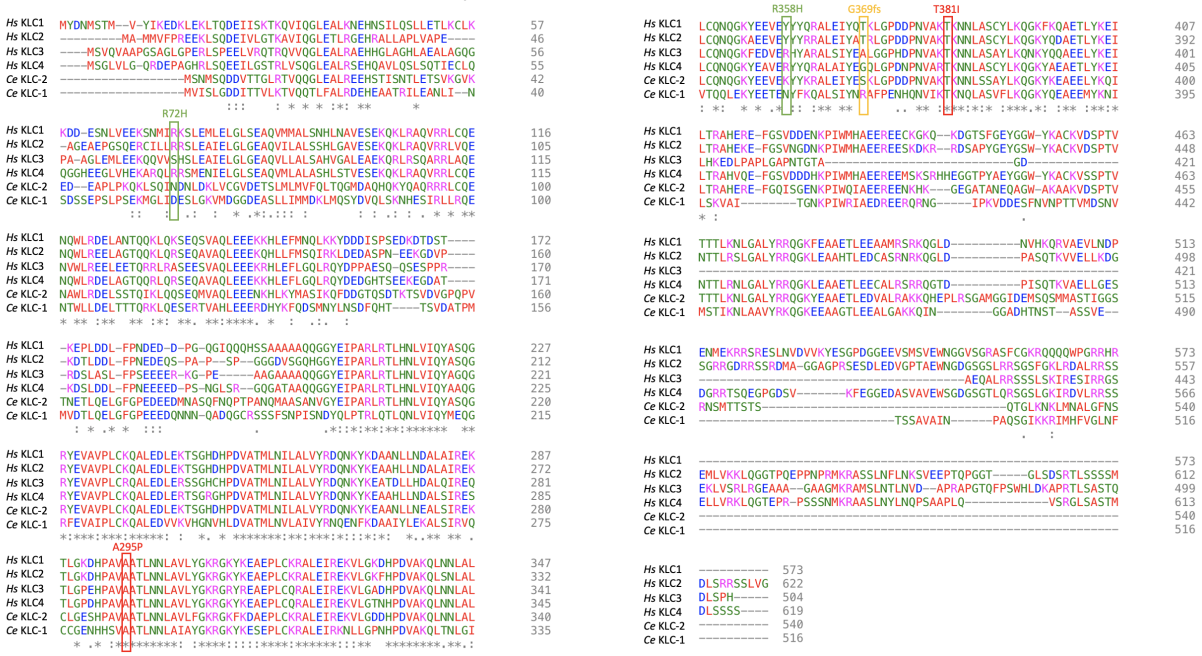

Fig. S1. Alignment of human and *C. elegans* kinesin light chains. A protein sequence alignment of the four human and two *C. elegans* kinesin light chains are shown. Amino acids are color coded based on side chain properties. The five residues introduced into the *hKLC4* line of *C. elegans* are boxed. R72H and R358H (green boxes) missense mutations are predicted to be benign. A295P and T381I (red boxes) missense mutations predicted to be pathogenic. G369fs (orange box) is the clinical variant of uncertain significance that introduces a frame shift and early stop. The notations underneath the residues show how conserved each residue is; . indicates at least 50% identity, : indicates a higher level of identity/similarity, and * indicates that the residue is identical in all 6 kinesin light chains.

TCAGCCATAAAATTGAAACTGGATAATATTTACCTAAATAATAGATTTTCCAAATTCTTGCACCAAATAGATCTATGGTATAAAACTGT
CAGGCTGATTTTCAAATTTTTGCATTTTTTAGAATTCCAAAGTGAGCAGTATTCATGGAAAAATATTTTTTGAATCTAGTTCTACGTTT
TTGAGAGAATGCAAGATTTTAATTAATTTTTGTTGTGCCATACTTTGGCATTATTTACATTTAAAAGAAAAAAAGGGTTAATTTTTTCT
GATAAATCTGAAATTAAAAAAAAAGACTATCGCAAAACTTATTGAACGACCTAATAGCTATGATCTACATAGATTCAACTGTTTTTACT
CACTGAAAAAATTTTCTCGTTAGCTACCAACACTTCTCGAACTTCTTCCAATTACATTTAATAGATTTAAAATTTCAGGCGAAAAATCG
AGTCGCTCGCGAAA**ATG**TCCGGACTCGTCCTCGGACAACGTGACGAGCCAGCCGGACACCGTCTCTCCCAAGAGGAGATCCTCGGATCC
ACCCGTCTCGTCTCCCAAGGACTCGAGGCCCTCCGTTCCGAGCACCAAGCCGTCCTCCAATCCCTCTCCCAAACCATCGAGTGCCTCCA
ACAAGGAGGACACGAGGAGGGACTCGTCCACGAGAAGGCCCGTCAACTCCGTCGTTCCATGGAGAACATCGAGCTCGGACTCTCCGAGG
CCCAAGTCATGCTCGCCCTCGCCTCCCACCTCTCCACCGTCGAGTCCGAGAAGCAAAAGCTCCGTGCCCAAGTCCGTCGTCTCTGCCAA
GAGAACCAATGGCTCCGTGACGAGCTCGCCGGAACCCAACAACGTCTCCAACGTTCCGAGCAAGCCGTCGCCCAACTCGAGGAGGAGAA
GAAGCACCTCGAGTTCCTCGGACAACTCCGTCAATACGACGAGGACGGACACACCTCCGAGGAGAAGGAGgtaagtttaaacatatata
tactaactaaccctgattatttaaattttcagGGCGACGCCACCAAGGACTCCCTCGACGACCTCTTCCCAAACGAGGAGGAGGAGGAC
CCATCCAACGGACTCTCCCGTGGACAAGGAGCCACCGCCGCCCAACAAGGAGGATACGAGATCCCAGCCCGTCTCCGTACCCTCCACAA
CCTCGTCATCCAATACGCCGCCCAAGGACGTTACGAGGTCGCCGTCCCACTCTGCAAGCAAGCCCTCGAGGACCTCGAGCGTACCTCCG
GACGTGGACACCCAGACGTCGCCACCATGCTCAACATCCTCGCCCTCGTCTACCGTGACCAAAACAAGTACAAGGAGGCCGCCCACCTC
CTCAACGACGCCCTCTCCATCCGTGAGTCCACCCTCGGACCAGACCACCCAGCCGTCGCCGCCACCCTCAACAACCTCGCCGTCCTCTA
CGGAAAGCGTGGAAAGTACAAGGAGgtaagtttaaacagttcggtactaactaaccatacatatttaaattttcagGCCGAGCCACTCT
GCCAACGTGCCCTCGAGATCCGTGAGAAGGTCCTCGGAACCAACCACCCAGACGTCGCCAAGCAACTCAACAACCTCGCCCTCCTCTGC
CAAAACCAAGGAAAGTACGAGGCCGTCGAGCGTTACTACCAACGTGCCCTCGCCATCTACGAGGGACAACTCGGACCAGACAACCCAAA
CGTCGCCCGTACCAAGAACAACCTCGCCTCCTGCTACCTCAAGCAAGGAAAGTACGCCGAGGCCGAGACCCTCTACAAGGAGATCCTCA
CCCGTGCCCACGTCCAAGAGTTCGGATCCGTCGACGACGACCACAAGCCAATCTGGATGCACGCCGAGGAGCGTGAGGAGATGTCCAAG
TCCCGTCACCACGAGGGAGGAACCCCATACGCCGAGTACGGAGGATGGTACAAGGCCTGCAAGgtaagtttaaacatgattttactaac
taactaatctgatttaaattttcagGTCTCCTCCCCAACCGTCAACACCACCCTCCGTAACCTCGGAGCCCTCTACCGTCGTCAAGGAA
AGCTCGAGGCCGCCGAGACCCTCGAGGAGTGCGCCCTCCGTTCCCGTCGTCAAGGAACCGACCCAATCTCCCAAACCAAGGTCGCCGAG
CTCCTCGGAGAGTCCGACGGACGTCGTACCTCCCAAGAGGGACCAGGAGACTCCGTCAAGTTCGAGGGAGGAGAGGACGCCTCCGTCGC
CGTCGAGTGGTCCGGAGACGGATCCGGAACCCTCCAACGTTCCGGATCCCTCGGAAAGATCCGTGACGTCCTCCGTCGTTCCTCCGAGC
TCCTCGTCCGTAAGCTCCAAGGAACCGAGCCACGTCCATCCTCCTCCAACATGAAGCGTGCCGCCTCCCTCAACTACCTCAACCAACCA
TCCGCCGCCCCACTCCAAGTCTCCCGTGGACTCTCCGCCTCCACCATGGACCTCTCCTCCTCCTCC**TGAATCTTCATTGTTGAGTTTAT**
CTTGTTGATTTTTGAATAAATTATCAACTCTTTACTTTTTAATGGGTTATGAAATAAATAAACATTGAAAACTGATAAACAACGTTCAT
CTCTGTTAACTCAAGGACTGGAGGACTTCTGGGGAGG**ATAACTTCGTATAGCATACATTATACGAAGTTAT**ATTTTTGCTTTCGTCGTA
AATCTACACACGCGTCTCTTCCGTGCGAGAGTCCAAGCCAGCAGCCAAATTCGTTGACTGAGTATTCAACGTTTATACGTTGTCGGCAA
CGAGAAATAGGAAAATGCATCGGGAAATGTTCTTTTTTCGATTTTTTCCAAGGTTTTGACAAATTTTACCACGAATTTTGCTATGTTTT
CAATTAAAAAATATGTTATTCAACTGTTTCTATGAGGAAAATAAGGCTTTGCATGTAATTTTCTTATTCAGCATAATTTTTAATTAATT
TGAATTTTCTGTCCTAACGTTTATTTTGTTTTCTTGGTTATGACTGATCTGAAATTAATTTTTGAATTTTAAGGTAATATGTCAGGCGG
TGCCGCAAGTTTGTACAAAAAAGCAGGCTCCATGAAAAAGCCTGAACTCACCGCGACGTCTGTCGAGAAGTTTCTGATCGAAAAGTTCG
ACAGCGTCTCCGACCTGATGCAGCTCTCGGAGGGCGAAGAATCTCGTGCTTTCAGCTTCGATGTAGGAGGGCGTGGATATGTCCTGCGG
GTAAATAGCTGCGCCGATGGTTTCTACAAAGATCGTTATGTTTATCGGCACTTTGCATCGGCCGCGCTCCCGATTCCGGAAGTGCTTGA
CATTGGGGAATTCAGCGAGAGCCTGACCTATTGCATCTCCCGCCGTGCACAGGGTGTCACGTTGCAAGACCTGCCTGAAACCGAACTGC
CCGCTGTTCTGCAGCCGGTCGCGGAGGCCATGGATGCGATCGCTGCGGCCGATCTTAGCCAGACGAGCGGGTTCGGCCCATTCGGACCG
CAAGGAATCGGTCAATACACTACATGGCGTGATTTCATATGCGCGATTGCTGATCCCCATGTGTATCACTGGCAAACTGTGATGGACGA
CACCGTCAGTGCGTCCGTCGCGCAGGCTCTCGATGAGCTGATGCTTTGGGCCGAGGACTGCCCCGAAGTCCGGCACCTCGTGCACGCGG
ATTTCGGCTCCAACAATGTCCTGACGGACAATGGCCGCATAACAGCGGTCATTGACTGGAGCGAGGCGATGTTCGGGGATTCCCAATAC
GAGGTCGCCAACATCTTCTTCTGGAGGCCGTGGTTGGCTTGTATGGAGCAGCAGACGCGCTACTTCGAGCGGAGGCATCCGGAGCTTGC
AGGATCGCCGCGGCTCCGGGCGTATATGCTCCGCATTGGTCTTGACCAACTCTATCAGAGCTTGGTTGACGGCAATTTCGATGATGCAG
CTTGGGCGCAGGGTCGATGCGACGCAATCGTCCGATCCGGAGCCGGGACTGTCGGGCGTACACAAATCGCCCGCAGAAGCGCGGCCGTC
TGGACCGATGGCTGTGTAGAAGTACTCGCCGATAGTGGAAACCGACGCCCCAGCACTCGTCCGAGGGCAAAGGAATAGATGCAAGATCC
TTTCAAGCATTCCCTTCTTCTCTATCACTCTTCTTTCTTTTTGTCAAAAAATTCTCTCGCTAATTTATTTGCTTTTTTAATGTTATTAT
TTTATGACTTTTTATAGTCACTGAAAAGTTTGCATCTGAGTGAAGTGAATGCTATCAAAATGTGATTCTGTCTGATGTACTTTCACAAT
CTCTCTTCAATTCCATTTTGAAGTGCTTTAAACCCGAAAGGTTGAGAAAAATGCGAGCGCTCAAATATTTGTATTGTGTTCGTTGAGTG
ACCCAACAAAAAGAGGAAACTTTATTGTGCCGCCAAGAAAAAAGTC**ATAACTTCGTATAGCATACATTATACGAAGTTAT**CCTCCCCAG
AAGTCCTCCAGTCCGCGGCCGCATCGTTCTTGTATTAATAAGTGCTCGTTGATTCAAGTTCCAAATTCTGAAATTTTGTCTCAGATCTA
CGAAAACTTGCTTTTTTCTTCTTTCGTTGTGTTGTGCATGATTCTTTTTTTTTTGATTGATTTTTCTTTCTGCCCCATCCCCTCATTCA
CAAAATCTTGTTCATAAATAATAGGGTTGAATAATTGAATGTTTCCTTGTCCGCCCCCACCAACTTTTTGGTAAAGCTTTATATTGTCT
GCTTCGAATTTTCTTTTCAAGCAAAAAAAAAAACATAATTATAACAAGCTGAAAAATCGTGTTGTTGTAAATAAAGGGTTTATTTTTGA
ATTCTGCTTGTTACAAGAAAAAGAAAACTTCAATTTCAGATGTCCGAATCTCGAAGAATGGAACGATCCGTTATGTATTGATTGTTGCT
CGACATTGTACTCCGTCATCAAGCTTTACACTTCTTCGCAAAATTCCAAAAGTTTACAAGTTCTCATTTATTTATATAAGCTTGGCG

Fig. S2. The *hKLC4* sequence and hygromycin resistance selection cassette inserted into the *klc-2* locus. Homology arms that are the native sequence of upstream and downstream of the *klc-2* open reading frame are shown in yellow. Codon-optimized *hKLC4* sequence is shown in black. Start and stop codons are bolded. Exons are shown in capital letters and synthetic introns are shown in lower-case letters. The hygromycin gene resistance selection cassette is shown in blue. The *eft-3* 3'UTR is bolded in blue. The loxP sites are bolded in purple. Everything between the loxP sites was removed before any analysis.

Disease Models & Mechanisms • Supplementary information

Table S1. C. elegans strains used in this study

**Strain**[1]	**Genotype**
N2	wild type
COP2335	*klc-2(knu1012[hKLC4::loxP::HygR::loxP]) V*
COP2355	*klc-2(knu1031[hKLC4]) V*
COP2396	*klc-2(knu1051[hKLC4(R358H)]) V*
COP2399	*klc-2(knu1054[hKLC4(T381I)]) V*
COP2401	*klc-2(knu1056[hKLC4(A295P)]) V*
COP2403	*klc-2(knu1058[hKLC4(R72H)]) V*
COP2460	*klc-2(knu1102[hKLC4(G369fs)]) V; Ex[klc-2::GFP]*
UD842	*ycIs9[pcol-10nls::gfp::LacZ] I; klc-2(knu1031[hKLC4]) V*
UD843	*ycIs9 I; klc-2(knu1051[hKLC4(R358H)]) V*
UD844	*ycIs9 I; klc-2(knu1058[hKLC4(R72H)]) V*
UD852	*ycIs9 I; klc-2(knu1056[hKLC4(A295P)]) V*
UD853	*ycIs9 I; klc-2(knu1054[hKLC4(T381I)]) V*
UD920	*ycIs9 I; klc-2(knu1102[hKLC4(G369fs)]) V; Ex[klc-2::GFP]*
UD393	*ycIs9[pcol-10nls::gfp::lacz]) I*
UD469	*ycIs10[pcol-10nls::gfp::lacz]) V*
Not named	*klc-2(km28) V; Ex[klc-2::GFP]*

[1]All strains except N2 (Brenner, 1974), the unnamed strain with *klc-2(km28)* and an extrachromosomal rescuing array (Sakamoto et al., 2005) (gift of Yishi Jin, University of San Diego), and the *ycIs9* and *ycIs10* strains (Bone et al., 2014) were created in this study.

Disease Models & Mechanisms • Supplementary information

Table S2. sgRNA and repair DNA templates used in this study

New Alleles[1]	sgRNA #1	sgRNA #2	Repair Template[2]
knu1012	CAGGCGAAAAA TCGAGTCGC	GGACTGGAGGA CTTCTGGGG	pNU2756[3]
knu1031	CTCAACAATGA AGATTCAGG	TTATTAATACAA GAACGATG	CTCCGCCTCCACCATGGACCTCTCCTCC TCCTCCTAAATAAATAAACTCGAGCAG GGTTATTGTCTCATGAGCGCACGTTCTT GTATTAATAAGTGCTCGTTGATTCAAG T
knu1051	GGTTTTGGCAG AGGAGGGCG	TTGTCCCTCGTA GATGGCGA	CACCCAGACGTCGCCAAGCAACTCAAC AACCTCGCTCTTCTTTGCCAGAATCAG GGAAAATACGAAGCTGTTGAGCACTAT TACCAGCGCGCTCTTGCCATCTACGAG GGACAACTCGGACCAGACAACCCA
knu1054	TTGTCCCTCGTA GATGGCGA	GCTTGAGGTAGC AGGAGGCG	CGAGGCCGTCGAGCGTTACTACCAACG TGCCCTCGCTATTTATGAAGGACAGCT TGGTCCAGATAATCCAAATGTTGCCCG CATCAAAAATAATCTTGCCTCCTGCTA CCTCAAGCAAGGAAAGTACGCCGAGG
knu1056	GTTTTGGTCAC GGTAGACGA	GCTTTCCGTAGA GGACGGCG	AGACGTCGCCACCATGCTCAACATCCT CGCCCTCGTTTATCGCGATCAGAATAA ATACAAAGAAGCTGCCCATCTCCTTAA TGATGCCCTTTCCATTCGTGAATCTACC CTTGGTCCAGATCATCCAGCTGTCCCA GCTACCCTTAATAATCTTGCCGTCCTCT ACGGAAAGCGTGGAAAGTACAAGGAG g
knu1058	TCCTTGTTGGA GGCACTCGA	CATCGAGCTCGG ACTCTCCG	CCAAGCCGTCCTCCAATCCCTCTCCCA AACCATCGAATGCCTTCAGCAGGGAGG TCATGAGGAAGGACTTGTTCATGAAAA AGCTCGCCAACTTCACCGTTCTATGGA AAATATTGAACTTGGACTTTCCGAGGC CCAAGTCATGCTCGCCCTCGCCTCCCA C
knu1102	ACGTTGGTAGT AACGCTCGA	GCTTGAGGTAGC AGGAGGCG	CCTCTGCCAAAACCAAGGAAAGTACGA GGCCGTCGAAAGATATTATCAGAGAGC TCTTGCTATTTATGAAGCAGCTGGAGC TGGACAGCCATAAGTTGCCCGCACTAA AAATAATCTTGCCTCCTGCTACCTCAA GCAAGGAAAGTACGCCGAGG

[1]All alleles are in *klc-2*.

[2]All the repair templates, except pNU2756[3], are single stranded DNA oligonucleotides. Blue text represents the sequence inserted between the cut sites of the two sgRNAs that has been recoded with synonymous codons. The purple text represents the codon of the introduced missense mutations.

[3]See Fig. S2 for the codon-optimized, artificial intron-containing, *hKLC4* sequence.

Disease Models & Mechanisms • Supplementary information

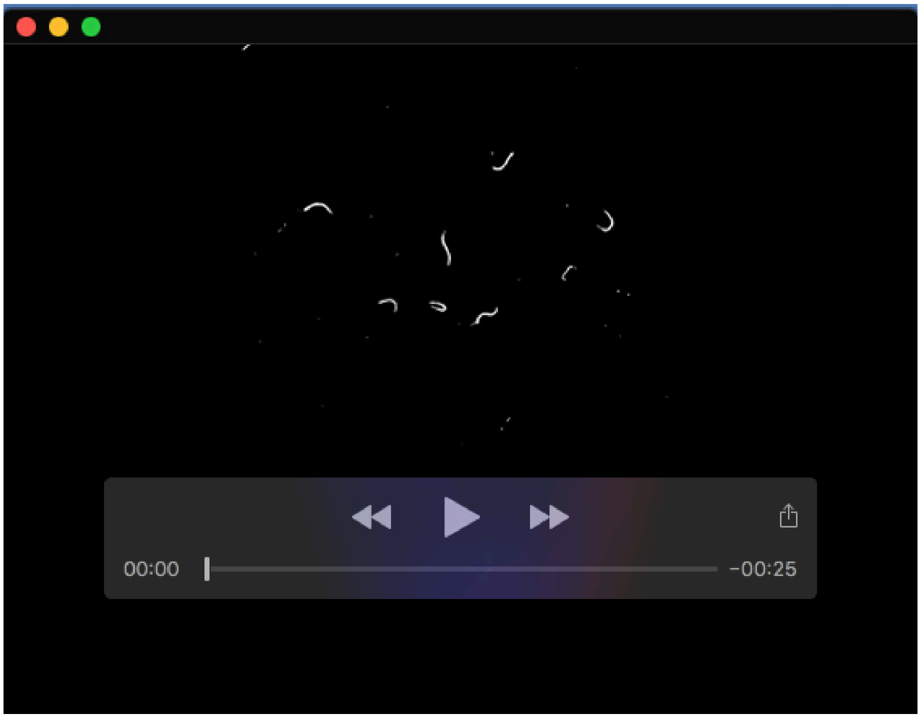

Movie 1. Wild type (N2) worms have no motility defects. L4-stage worms swimming in buffer. Fiji wrMTrck plugin (Nussbaum-Krammer *et al.,* 2015) was used to track each worm to measure the number of body bends per second (BBPS).

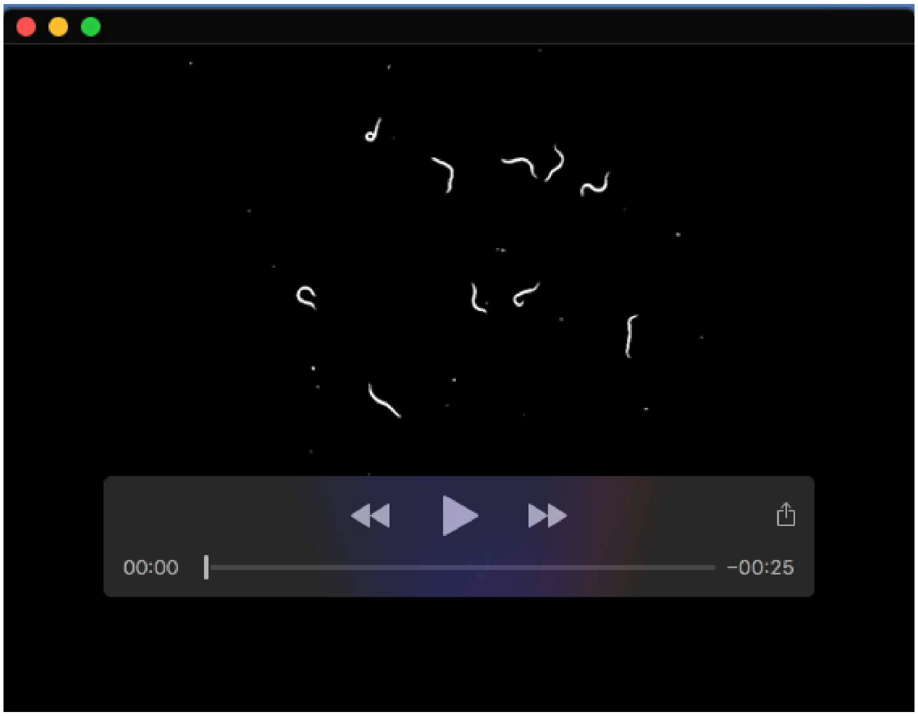

Movie 2. *hKLC4* worms have a significant motility defect, but they retain most of their swimming ability. L4-stage worms swimming in buffer. Fiji wrMTrck plugin(Nussbaum-Krammer *et al.,* 2015) was used to track each worm to measure the number of body bends per second (BBPS).

Disease Models & Mechanisms • Supplementary information

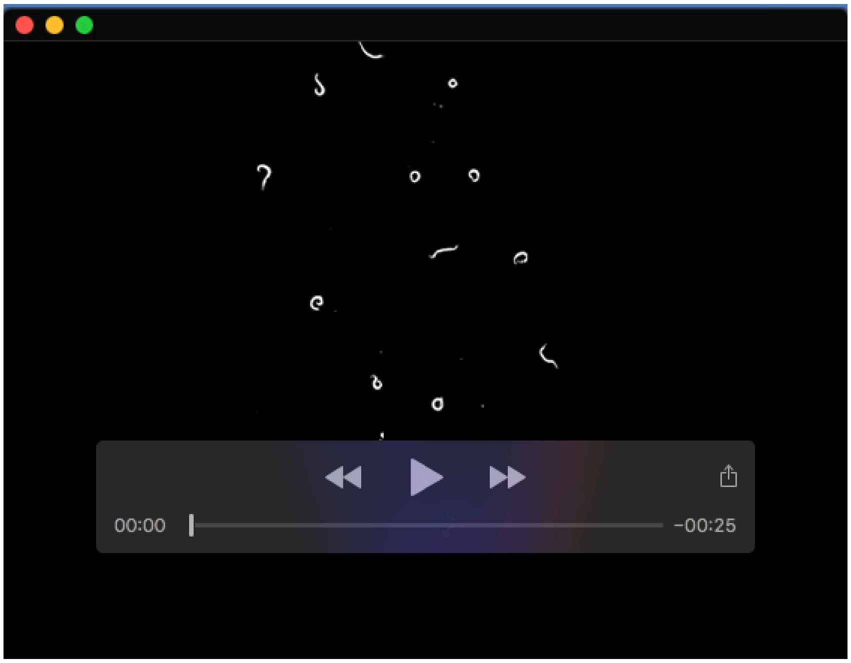

Movie 3. *hKLC4* T381I worms have a severe motility defect. L4-stage worms swimming in buffer. Fiji wrMTrck plugin(Nussbaum-Krammer *et al.,* 2015) was used to track each worm to measure the number of body bends per second (BBPS).

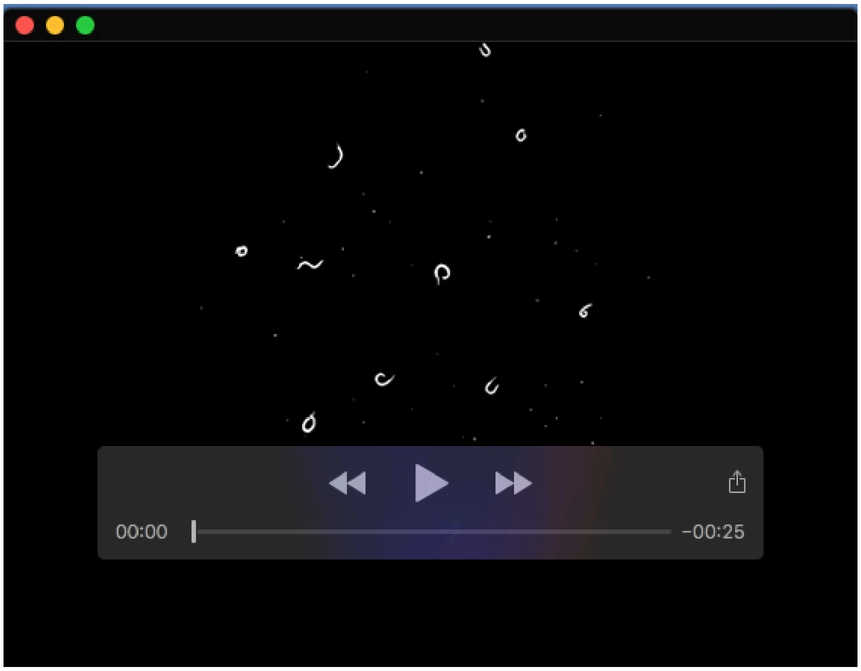

Movie 4. *hKLC4* T381I/+ worms have a severe motility defect. L4-stage worms swimming in buffer. Fiji wrMTrck plugin(Nussbaum-Krammer *et al.,* 2015) was used to track each worm to measure the number of body bends per second (BBPS).

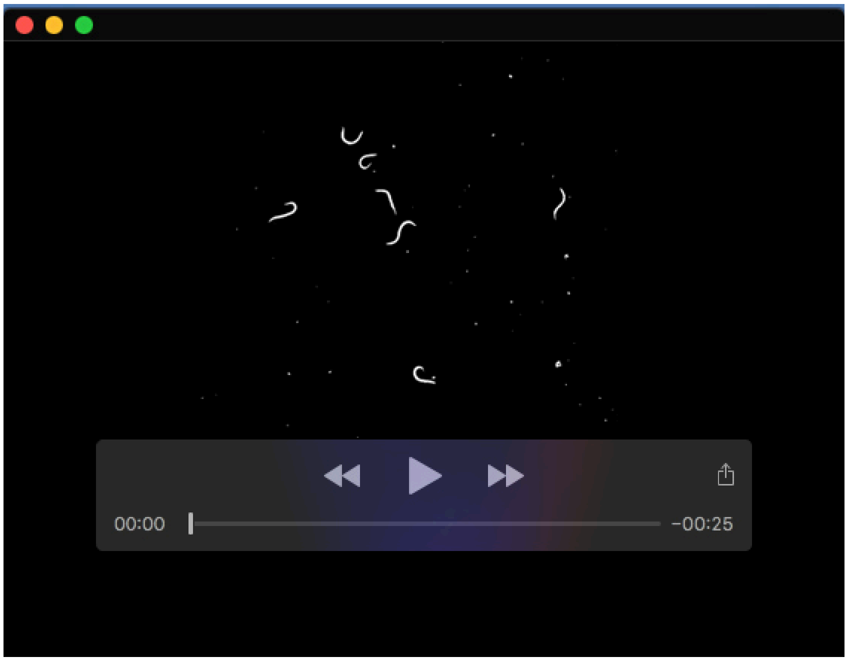

Movie 5. *hKLC4* G369fs/+ worms have a significant motility defect, but they retain most of their swimming ability. L4-stage worms swimming in buffer. Fiji wrMTrck plugin(Nussbaum-Krammer *et al.,* 2015) was used to track each worm to measure the number of body bends per second (BBPS).
